# Toward Gene-Correlated Spatially Resolved Metabolomics with Fingerprint Coherent Raman Imaging

**DOI:** 10.1021/acs.jpcb.3c01446

**Published:** 2023-06-13

**Authors:** Rajas Poorna, Wei-Wen Chen, Peng Qiu, Marcus T. Cicerone

**Affiliations:** † Department of Chemical and Biomolecular Engineering, Georgia Institute of Technology, Atlanta, Georgia 30332, United States; ‡ Department of Chemistry, Georgia Institute of Technology, Atlanta, Georgia 30332, United States; § Department of Biomedical Engineering, Georgia Institute of Technology, Atlanta, Georgia 30332, United States

## Abstract

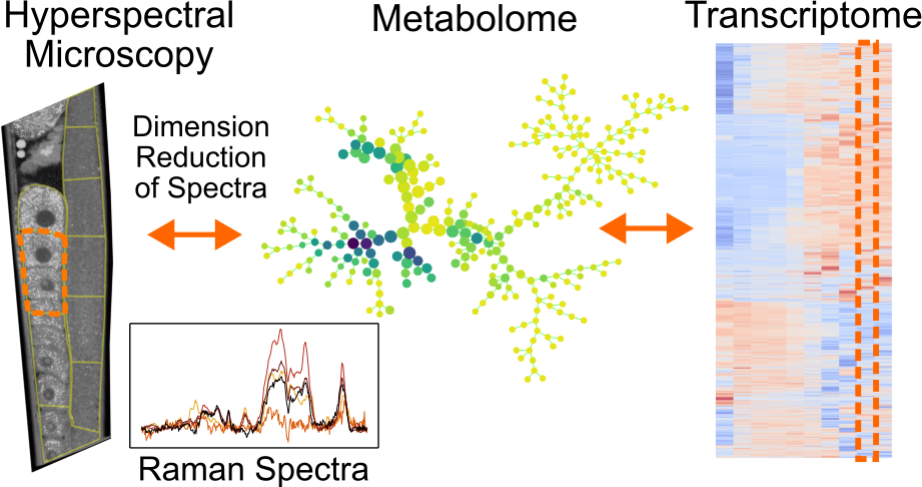

Raman spectroscopy has long been known to provide sufficient information to discriminate distinct cell phenotypes. Underlying this discriminating capability is that Raman spectra provide an overall readout of the metabolic profiles that change with transcriptomic activity. Robustly associating Raman spectral changes with the regulation of specific signaling pathways may be possible, but the spectral signals of interest may be weak and vary somewhat among individuals. Establishing a Raman-to-transcriptome mapping will thus require tightly controlled and easily manipulated biological systems and high-throughput spectral acquisition. We attempt to meet these requirements using broadband coherent anti-Stokes Raman scattering (BCARS) microscopy to spatio-spectrally map the *C. elegans* hermaphrodite gonad *in vivo* at subcellular resolution. The *C. elegans* hermaphrodite gonad is an ideal model system with a sequential, continuous process of highly regulated spatiotemporal cellular events. We demonstrate that the BCARS spatio-spectral signatures correlate with gene expression profiles in the gonad, evincing that BCARS has potential as a spatially resolved omics surrogate.

## Introduction

Omics approaches characterize aspects of cell or tissue composition at a level sufficient to discriminate among similar but functionally distinct phenotypic states. Methods used in such studies are often expensive, time-consuming, and destructive. In principle, similar information could be acquired quickly and noninvasively by quantifying chemical components in the sample via vibrational spectroscopies such as infrared absorption or Raman scattering. In practice, label-free vibrational spectroscopy methods do not have the specificity to distinguish among the lowest-abundance components of biological systems, such as signaling proteins or low-concentration metabolites, nor can they discriminate molecular sequences in proteins or nucleic acids. However, they can be very effective for classifying phenotypes based on robust but sometimes subtle aspects of chemical profiles.

Vibrational spectra report on *all* chemical species present at moderate and high concentrations through intrinsic vibrational resonances. With a reasonable spectral resolution (5–10 cm^–1^), ∼45 chemical bond-specific peaks can be identified in the “fingerprint” spectral region (from 500 to 1800 cm^–1^) of a Raman spectrum taken from biological cells.^1^ These peaks are relatively weak but highly informative. Another five, much (10×–100×) stronger peaks are found in the “CH stretch” region (from 2800 to 3000 cm^–1^). Using only the fingerprint spectra and assuming a 3:1 signal-to-noise ratio, such a spectrum can encode 3^45^ ≈ 10^21^ distinct states. With sufficient sensitivity to chemical changes, this specificity is ample to identify subtle phenotype changes. Indeed, individual Raman spectra, with acquisition averaged over entire cells, have been used to distinguish differentiated and pluripotent cells,^2−5^ to identify activated immune cells,^6−8^ and to discriminate between bacterial subtypes.^9−11^ Even at a spectral resolution of 30 cm^–1^, rapid bacterial phenotype sorting is possible.^11^

The ability to discriminate between phenotypes without fully profiling *all* chemical components results from the feedback-controlled nature of biological systems. Despite their complexity, these highly constrained systems occupy small portions of their available phase space. The dense, feedback-controlled regulatory networks that control biological function lead to state functions with a relatively small number of “basins of attraction” (phenotypes).^12^ The resulting relatively small number of phenotypes have discrete overall chemical profiles.

The chemical profiles that characterize these attractor states are direct downstream results of gene regulation activity. While the connection between individual gene activation and metabolite production is not always unique,^13^ overall chemical profiles associated with gene family activity will correlate with Raman spectra. Recently, Kobayashi-Kirschvink et al.^14^ have shown a strong correlation between transcriptomic profiles and single-cell Raman spectra and were able to identify anchor gene activation from changes in Raman spectra for phenotypes related to those on which their model was trained.

To the extent that the connections between Raman spectra and signaling profiles are unique and measurable, Raman spectroscopic imaging may become a powerful tool for rapidly profiling the activity of gene families, even with subcellular resolution. Further, it may be possible to acquire Raman spectra noninvasively, facilitating longitudinal studies of individual cells or living organisms. Spontaneous Raman scattering is weak, making imaging difficult without parallel acquisition.^15^ Coherent Raman imaging (CRI) methods shorten the time needed for acquiring the Raman signal.^20^ However, most CRI techniques acquire the Raman signal only in a narrow portion of the strong CH stretch region. A few CRI studies^17−19^ have acquired a moderate spectral range (usually <400 cm^–1^) in the fingerprint region. However, full spectral imaging using these methods is prohibitive.^20^ Unlike most coherent Raman approaches, broadband coherent anti-Stokes Raman scattering (BCARS) can rapidly and reliably acquire fingerprint and CH-stretch Raman spectra. Further, the signals are linear with analyte concentration and intrinsically calibrated due to their interaction with the electronic nonresonant background.^20^

**Figure 1:**
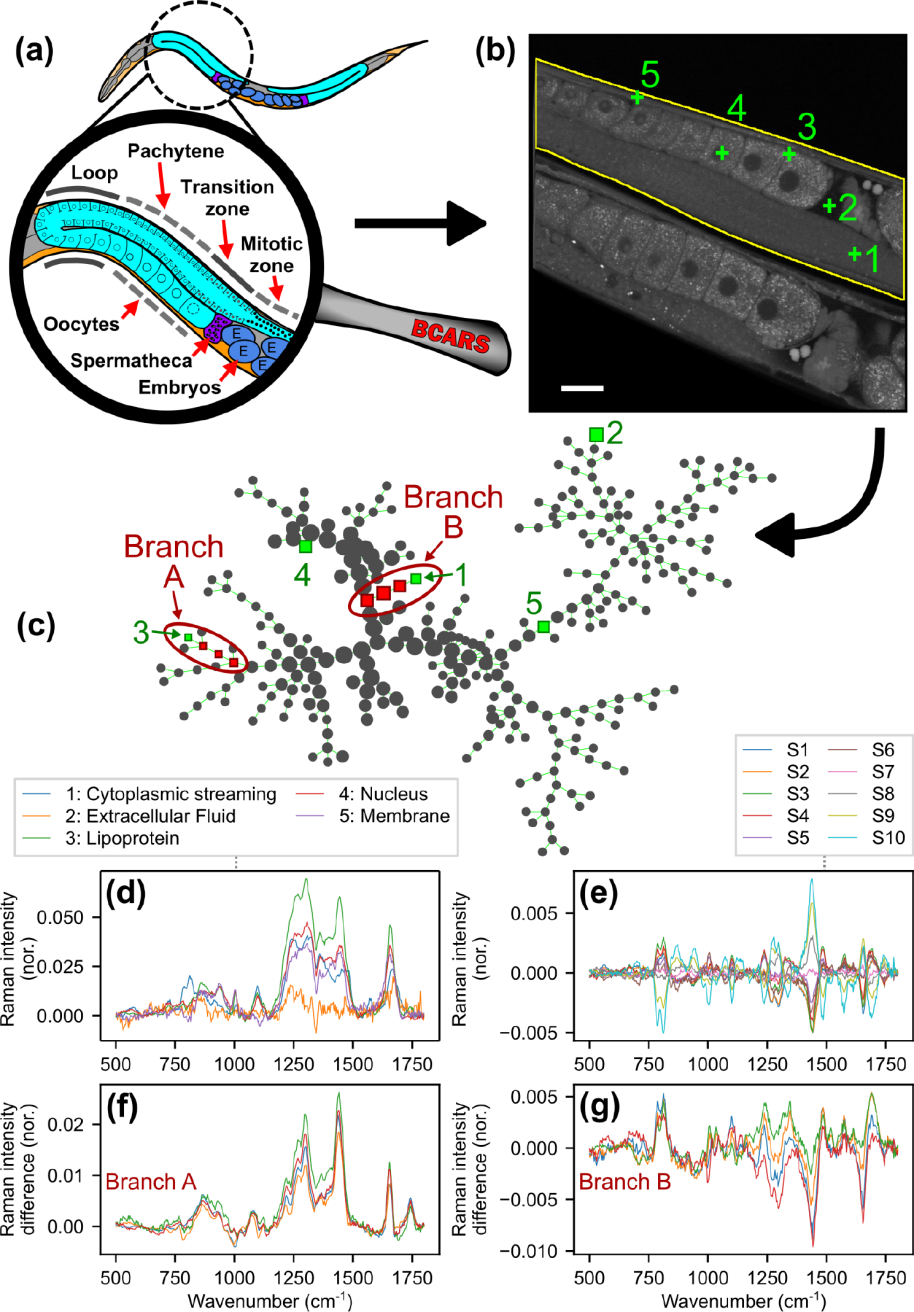
BCARS imaging of *C. elegans* gonad and SPADE analysis. (a) Schematic drawing of a *C. elegans* hermaphrodite reproductive system. (b) 2925 cm^–1^ BCARS image of two wild-type adult worms. Each pixel spectrum was acquired in 4 ms. The yellow line indicates the outline of the upper worm (“Up”). Scale bar: 20 μm. (c) SPADE tree representation of fingerprint BCARS spectra from the outlined area. Pixels 1–5 shown in (b) belong to the indicated nodes on the SPADE tree. (d) BCARS spectra corresponding to the 5 pixels, with their identities labeled in the legend. (e) Spatially averaged difference spectra of the 10 gonad sections of Up indicated in S1, with the mean spectrum of the entire Up gonad being subtracted. (f, g) Difference spectra corresponding to each node in branches A and B, respectively, labeled in (c). The mean spectrum of the full Up worm is subtracted. Note that the difference spectra are similar within a branch, but completely different across branches.

As a starting point for investigating the possibility of using the quantitative Raman features retrieved by BCARS to convey spatially resolved metabolomic information in intact living systems, we chose a widely used model animal *Caenorhabditis elegans* (*C. elegans*). Because of its optically transparent body, well-defined anatomy, and genetic similarity with humans, *C. elegans* is an excellent experimental model for the studies of signaling pathways,^21^ aging,^22^ development,^23^ and reproduction.^24^ Of particular interest for the present study, the *C. elegans* gonad is a precise and highly regulated system. Because the turnover time of the entire volume of the gonad is short, about 6.5 h,^25^ all of the critical cellular events, including germline stem cell proliferation and differentiation, meiotic maturation, meiotic chromosome reorganization, oocyte growth, and ovulation, are highly coordinated to ensure successful fertilization.^26,27^ This unique model system offers a great opportunity to study various temporally and spatially progressing cell types from the undifferentiated proliferative germ cells in the mitotic zone, cells entering meiotic prophase in the transition zone, cells undergoing prophase of meiosis, the pachytene stage, programmed cell death in the zones of pachytene and loop, and finally to maturing oocytes (Figure 1a) within a single gonad tissue.

In this study, we compare spectroscopic BCARS images of *C. elegans* gonad to data from a spatially resolved transcriptomic profile of the gonad in wild-type animals.^28^ While the spatial gene expression study employed delicate isolation and dissection of *C. elegans* gonad, dividing the whole gonad tissue into 10 sections,^28^ BCARS offers a subcellular spatial resolution. At this resolution, BCARS can capture metabolomic information in live, intact animals. Here, we have used SPADE (Spanning-tree Progression Analysis of Density-normalized Events)^29^ as a tool for characterizing Raman spectra and quantitatively correlating their changes with transcriptomic data. Other methods, such as UMAP,^30^ may also be suitable for our purposes, but here we focus on a demonstration of principle; optimization of analytic methods will follow in later work. In this work, we demonstrate a strong correspondence between Raman spectral changes and transcriptomic profiles in the *C. elegans* gonad. We demonstrate that it may be possible to spatially map gene regulation at a subcellular level using spectroscopic Raman imaging.

## Materials and Methods

### BCARS

Inspired by the pioneering work of Zumbusch and Xie,^31^ we introduced broadband coherent anti-Stokes Raman scattering (BCARS) as a spectroscopic coherent Raman imaging modality in 2004.^32^ The approach is described in detail elsewhere.^20,33^ Briefly, BCARS uses impulsive excitation from a near-infrared continuum pulse of ∼2000 cm^–1^ bandwidth (∼900–1350 nm) and ∼16 fs duration to generate coherence in the fingerprint region. Coherence in the CH stretch region is generated by interactions between the broad continuum pulse and a spectrally narrow, ≤10 cm^–1^ bandwidth and temporally long, ∼3 ps probe pulse at 770 nm. The pulse repetition rate for both pulses is 40 MHz. The two beams were collinearly aligned and focused onto the sample by a 60× water immersive objective (UPlanSApo, Olympus, Japan) with an average power after objective of 5 and 9.6 mW for the continuum and ps probe, respectively. Under such conditions, the diffraction-limited diameter of the focal spot for the two beams is ∼650 nm (continuum) and ∼500 nm (ps probe). The energy per pulse is 0.12 nJ (continuum) and 0.24 nJ (ps probe), respectively. Probe pulse photons inelastically scatter from the coherence generated, and the anti-Stokes signal is simultaneously generated at all Raman shifts up to 3500 cm^–1^ at each laser shot. The signal is collected in the forward scattering direction and dispersed on a spectrograph. This scheme enables rapid spectral acquisition (∼4 ms/pixel) covering the fingerprint and CH stretch^33^ at optical resolution (∼0.5 μm). Both images in this work have a resolution of 660 × 660 pixels (200 × 200 μm^2^), leading to an overall acquisition time of ∼29 min per image. In spite of the efficient coherence generation mechanism for the fingerprint region, the spectral intensity there is quite small. BCARS uses the nonresonant background (NRB) as a local oscillator to amplify, linearize, and normalize the weak fingerprint signal.^20^

Ironically, the NRB had been universally viewed as a nuisance and caused many to abandon CARS for SRS.^34^ When used properly, the NRB simultaneously acts as a robust amplifier of small signals and as an internal reference, yielding Raman spectra with intrinsic calibration and absolute peak heights and ratios.^20^ Briefly, the Kramers–Kronig (KK) transform is used to extract a phase term from each measured pixel spectrum. This phase term consists of a slowly varying term from the NRB and a quickly varying term from the Raman resonances. With a reference NRB from water or glass, most of the NRB phase can be removed across the image. The remaining part due to variation of the NRB across the sample can be removed using standard per-pixel baseline correction techniques. An inverse transform uses the ratio of the Raman peak height to the NRB to return the calibrated, amplitude-standardized Raman spectrum. Consequently, with BCARS we obtain Raman spectra that, in principle, do not vary day-to-day or even between instruments.

### Strains and Reagents

The wild-type *C. elegans* (Bristol N2) worms used in this study were obtained from the Caenorhabditis Genetics Center (CGC), University of Minnesota. Nematodes for measurements were synchronized by the two-generation egg-laying method. Synchronized worms were maintained on nematode growth media (NGM) plates, seeded with *Escherichia coli* strain OP50 as a bacterial food source, and incubated at 20 °C until 1 day adults. Sodium azide (Sigma-Aldrich) was used to anesthetize worms for microscopy.

### Spatial Segmentation of Worms

Because we compare our spectral data to spatial transcriptomic data of Tzur et al.,^28^ we needed to coregister our data. Tzur et al. performed microsurgery on the worms, excising the worm gonad and cutting it into 10 equal-length sections. We segmented our worm images to correspond with the sectioning choices made by Tzur et al.,^28^ as shown in Figure S1. The number of spectra used for analysis (per gonad section per worm) is listed in Table S1.

### SPADE Algorithm

SPADE is described in detail elsewhere.^29^ As applied here, SPADE takes as input the BCARS spectrum of each pixel (651 numbers per pixel: one value for every 2 cm^–1^ from 500 to 1800 cm^–1^), treated as a vector, and calculates the density of points in this 651-dimensional vector space. We call this vector space the “spectral space” and define the density of points as the “spectral density”. SPADE then downsamples the data in a density-dependent manner, so that the density of points in the downsampled data in spectral space is approximately uniform in the volume spanned by the data. Where points constitute a density lower than a set threshold (1 percentile), they are treated as outliers and removed. Then, SPADE performs either K-means or agglomerative clustering to partition the downsampled data into a user-defined number of clusters. Next, a minimum spanning tree (MST) is constructed to connect the clusters according to their similarity. Finally, the MST is displayed using a force-directed drawing algorithm. The node connectivity conveys spectral similarity information; aside from constraints due to this connectivity, the *x*–*y* coordinates of the nodes convey no information.

Because SPADE was developed for flow cytometry data analysis, the BCARS data here was exported into Flow Cytometry Standard (FCS) files accepted by the MATLAB implementation of SPADE (available online^35^). Each pixel was treated as a “cell” in FCS, with 651 “markers” corresponding to each wavenumber in spectral space. Two SPADE trees were constructed here: one called “Full” and the other called “Gonad”. Full was constructed using all pixels belonging to the “Up” worm as defined by the mask shown in Figure S1. Gonad was constructed using only pixels belonging to the gonad of the Up worm. All the SPADE parameters for both trees were the same. The downsampling step required a user-defined target density, which dictated the desired density of points after downsampling, and was set to be the third percentile of the local density of all data points. The maximum number of allowable cells in the pooled downsampled data was set to 2 × 10^5^, greater than the number of pixels supplied. The resulting downsampled data were clustered using K-means to generate 300 clusters, each of which was represented by one tree node. Each pixel has an *X* and *Y* position in the image data, which was included in the FCS file but ignored while building the SPADE tree. Once the clustering and MST construction were performed to generate the SPADE tree, the position information associated with the pixels was used to highlight pixels in the BCARS image corresponding to the nodes of interest.

Overall, SPADE constructs a tree to summarize and visualize the heterogeneity of pixels in the spectral data. Each pixel is assigned to exactly one node, but each node may have many pixels mapped onto it if the pixels all have similar spectra. The node association and image location data are retained together, but the latter is not used to generate the tree; it is however, retrieved to highlight image pixels corresponding to particular nodes.

Figure 3 illustrates how nodes of the SPADE tree are colored to visualize the spectral heterogeneity in various regions of the image. Contrast for each of the 10 trees is derived from the data of the corresponding gonad section. Nodes are colored according to the fraction of image pixels assigned to them in the corresponding gonad section. The darkest nodes have the highest percentage of pixels in their gonad section.

### Correlation and Clustering Algorithms

All correlations were calculated using Pearson product-moment correlation coefficients. To generate Figure 4c, the correlation between each pair of the 300 Gonad tree nodes across the 10 sections (a 300 × 300 matrix) was supplied to Seaborn’s clustermap function, which performs a hierarchical clustering of this matrix (Figure S2) resulting in a new ordering for the nodes. For each node, the *z*-score (subtract mean, divide by variance) of its fraction in each of the 10 sections was computed. The *z*-score for each node was displayed according to the new hierarchical ordering supplied by clustermap. For Figure 4d, the same procedure was followed with the data in Tzur et al.’s S3 table,^28^ which includes data for “dynamic” genes, whose expression changes by at least 2-fold within the gonad (Figure S3).

### Software Versions

We use the following software versions: Python 3.7.5, Seaborn 0.12.1, NumPy 1.21.6, Pandas 1.3.5, CRIkit2 0.4.4, MATLAB R2019a and R2022b, UMAP 0.5.3, and SPADE V3.0 with some UI, visualization, and export customizations but no data handling modifications.

## Results

### Representing BCARS Spectral Density as a Tree

Figure 1a shows a schematic representation of the *C. elegans* gonad. Figure 1b shows a BCARS hyperspectral image of two worms lying side by side, which we analyze in this work. Here and below, we refer to the upper worm as “Up” and to the lower worm as “Down”. Each pixel contains a complete Raman spectrum from 500 to 3400 cm^–1^, of which we only use 500 to 1800 cm^–1^ in this analysis. Figure S4a–c shows the same image visualized at different wavenumbers. The spectra of five distinct points marked in Figure 1b are shown in Figure 1d. The spectra show distinct and meaningful changes. To highlight these changes, we will, in some cases, present “difference spectra”, where we subtract an appropriate spatially averaged mean spectrum.

Tzur et al.^28^ divided the worm gonad into 10 linear sections to perform transcriptomics. We section our worms correspondingly, as shown in Figure S1. In Figure 1e, the mean Raman spectrum of the entire worm gonad visible in Figure 1b was subtracted from the mean Raman spectrum of each of the 10 sections. Figure 1e shows these difference spectra.

Raman fingerprint spectra of biological systems, as shown in Figure 1d, typically exhibit ∼45 peaks. As mentioned above, this leads to an information phase space with 10^21^ discernible configurations. The possibility of small peak shifts from different local molecular environments further augments the effective information content. Position, amplitude, and width information for each peak is encoded at a slightly oversampled two-wavenumber resolution over the range 500–1800 cm^–1^ (the spectral resolution is 10 cm^–1^). Treating each spectrum as a 651- dimensional vector, each pixel becomes a point in a high-dimensional vector space. A hyperspectral image can thus be treated as a distribution of points in this space.

Interpreting the spectral distribution or density directly can be cumbersome without dimension reduction and visualization tools. We expect that the distribution in a BCARS image is sparse; i.e., most of the 651-dimensional vector space is unoccupied. Rather, most spectra should fall into distinct clusters in this space because the overall chemical compositions they report on are similar across cells. Some spectral data will fall into intermediate points between these clusters because some pixels will be at the boundary between two regions of well-defined chemical composition. Such a distribution suggests a potential visualization: a network of nodes representing these clusters and intermediate points. SPADE is an algorithm that implements this visualization by identifying clusters, computing their connectivity using a minimum-spanning tree, and displaying the tree using force-directed drawing (see the Materials and Methods section for more details).

Figure 1c shows a SPADE tree constructed using spectra from the entire Up worm, outlined in yellow in Figure 1b. Each pixel is assigned a node. The nodes corresponding to each of the five pixels marked in Figure 1b are highlighted in green. The tree clusters spectra into nodes, and nodes into branches, both by similarity. Therefore, the pixels that belong to a node should have similar spectra. We define the spectrum of a node as the mean spectrum of all pixels assigned to that node. We can further expect that nodes within a branch should contain similar but slightly different spectra and that different branches should show greater variation in spectra, depending on the distance between the branches. This expected pattern is demonstrated in Figure 1f,g, which shows difference spectra of each selected node in branches A and B, with the mean spectrum of the full worm subtracted.

This representation allows for robust interpretation even in the presence of spectral variations due to the changing environment in different tissues. These environmental changes could induce shifts or broadening in characteristic peaks of important biomolecules. Further, while our algorithm corrects for the variations in NRB across the tissue,^20^ subtle, systematic variations in important peaks due to different local environments may still be present in the processed spectra. However, such significant shifts in environmental composition are likely to also lead to overt differences in the pixel spectrum. SPADE assigns different nodes to pixels with systematically altered spectra. Thus, pixels from significantly different environments will show up on the SPADE tree as different nodes or branches.

### Spectral and Spatial Analysis of BCARS Metabolomic Data

**Figure 2:**
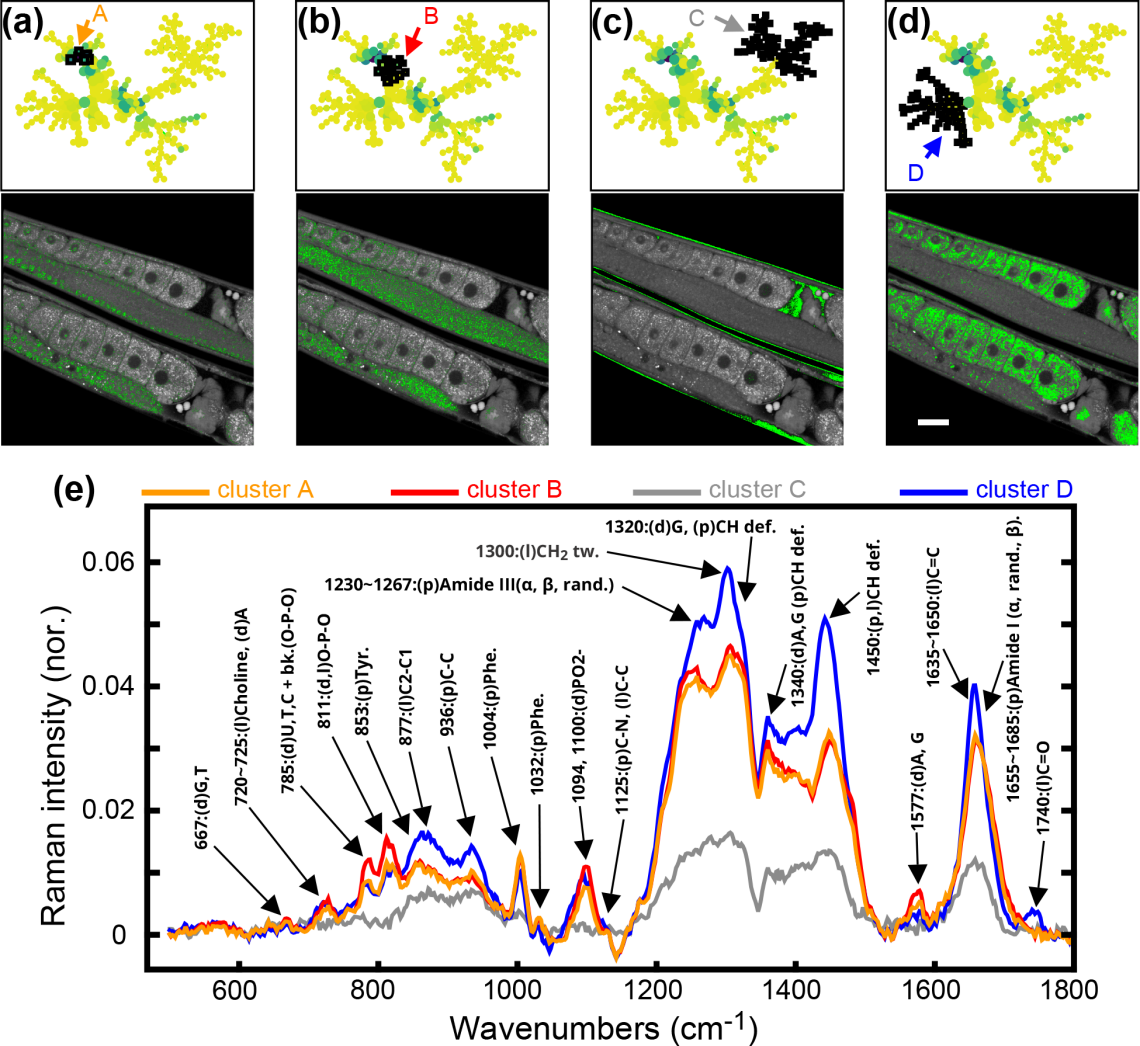
Clusters of nodes and their spatial distribution in the 2925 cm^–1^ BCARS image and the corresponding BCARS spectra. (a–d) Different clusters of nodes (black squares) and the corresponding spatial distribution (green dots) in the BCARS image. Scale bar: 20 μm. (e) Raman spectra of clusters A–D shown in (a–d), and the assignment of related Raman peaks. d, DNA/RNA or nucleic acids; l, lipid; p, protein.

SPADE sorts BCARS spectra in an unsupervised, hierarchical way and connects branches and leaf nodes within a tree. The connectivity of these nodes provides a measure of the similarity of biologically and functionally related components with slightly different chemical compositions. We highlight this in Figure 2. Selecting the two neighboring node clusters, A and B, from Figure 2a,b, within a SPADE tree that was generated using the whole-worm area in the BCARS images, we found their BCARS spectra were similar (Figure 2e), but their spatial distribution in the gonad was different. While cluster A pixels were mostly located in the nuclei of the germ cells in the mitotic zone, transition zone, pachytene, loop, and in some premature oocytes (Figure 2a), cluster B highlighted the core of gonad (Figure 2b) with a distribution identical with cytoplasmic streaming, a fluidic force in the gonad involved in transporting nutrients, filaments, mRNA, and other materials toward the proximal gonad end and into enlarging oocytes.^36^ Compared to the position of cluster A in the BCARS SPADE tree, cluster B is closer to the center consistent with the fact that the chemical makeup of nuclei in the developing germ cells (cluster A) originated from the cytoplasmic streaming (cluster B).

Different branches highlighting the pixels with similar spatial distribution could be chemically distinct. We selected two clusters of nodes within completely different branches (clusters i and ii in Figure S6). The corresponding pixels of these two clusters are mostly distributed in the nuclei of oocytes and epidermal cells. However, their BCARS spectra show a significantly different intensity level for nucleotide-related bands at 785 cm^–1^ (O–P–O nucleic acid backbones), 811 cm^–1^ (O–P–O), and 1340 cm^–1^ (A, G) as well as other protein- or lipid-related bands at 877 cm^–1^ (l, C–C), 936 cm^–1^ (p, C–C), 1230–1267 cm^–1^ (p, amide III), 1300 cm^–1^ (l, CH_2_ tw), and 1450 cm^–1^ (p and l, CH def) (Figure S6). While both DNA and RNA have peaks at 785 cm^–1^, only RNA has a strong peak at 811 cm^–1^,^37−45^ and thus, the intensity ratio between 811 and 785 cm^–1^ peaks provides a measure of the RNA abundance in the pixel spectrum. Compared to cluster i, the 811 cm^–1^/785 cm^–1^ ratio is higher in cluster ii, suggesting more RNA components in the (cluster ii)-highlighted region (Figure S6). Other branches, such as clusters C and D shown in Figure 2c–e, simply show significantly different spatial patterns and BCARS spectra. Cluster C pixels are mostly the extracellular fluid in the body cavity without obviously strong Raman peaks in the fingerprint region. The average BCARS spectrum of cluster D shows strong lipid and protein bands at 877 cm^–1^ (l, C–C), 936 cm^–1^ (p, C–C), 1004 cm^–1^ (p, phe), 1230–1267 cm^–1^ (p, amide III), 1300 cm^–1^ (l, CH_2_ tw), 1450 cm^–1^ (p and l, CH def), 1635–1650 cm^–1^ (l, C=C), and 1740 cm^–1^ (l, C=O), which is in agreement with our previous BCARS measurement of yolk lipoprotein complex,^46^ an apolipoprotein-B-like complex that brings lipids and nutrients from the intestine to developing oocytes/embryos,^47^ indicating most of those highlighted pixels in the oocytes and embryos are essentially in the yolk granules. These demonstrate that the chemical and spatial information captured by BCARS is biologically meaningful, providing insights into the underlying functional role of observed objects.

### Comparison between BCARS Metabolomic Data and Gene Expression Profile along the Hermaphrodite Gonad

**Figure 3:**
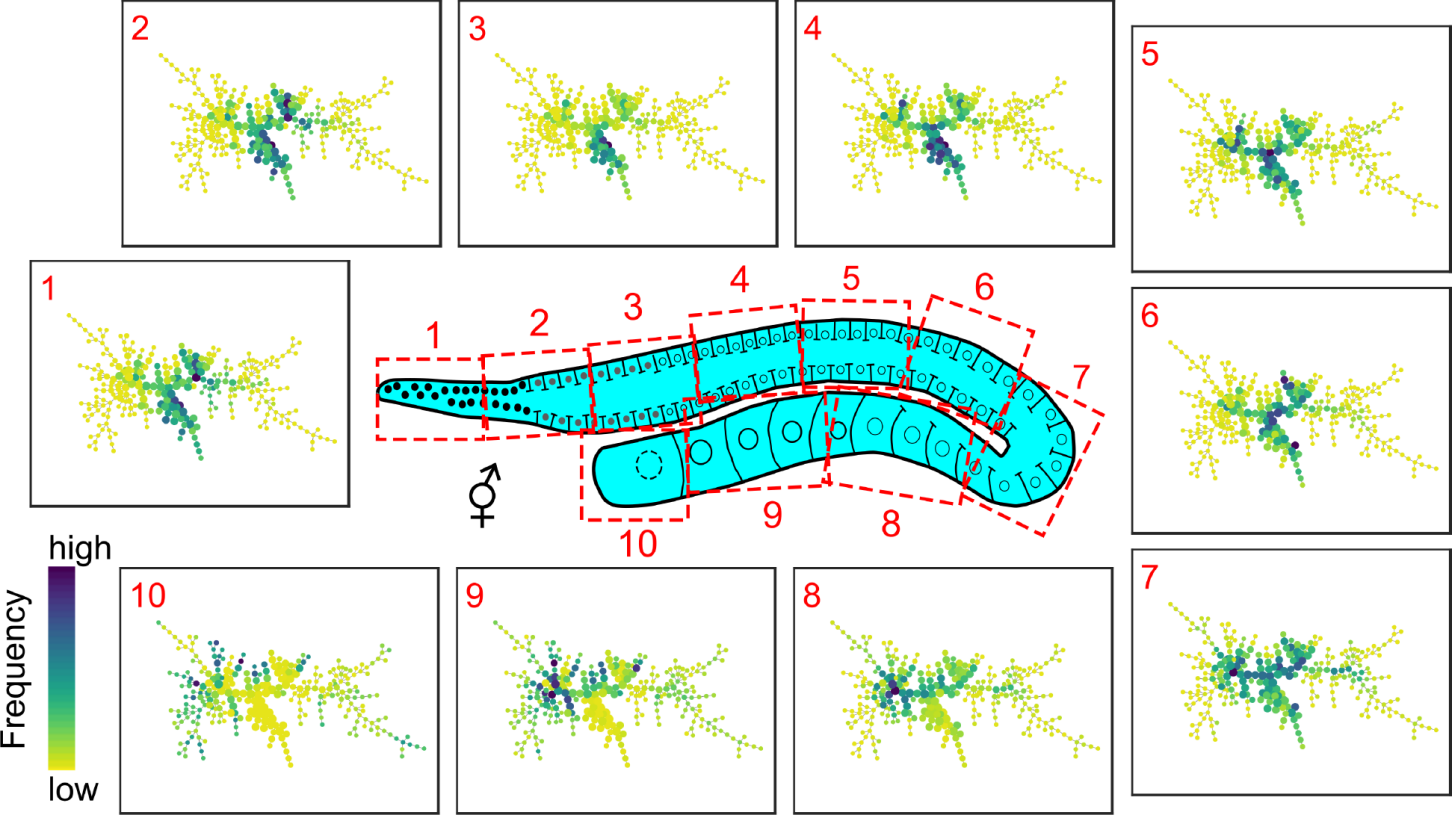
BCARS SPADE tree plot for gonad sections. The *C. elegans* gonad arm imaged by BCARS was divided into 10 sections with the same spatial segmentation method described in ref (28) (see the Materials and Methods section for details). For each section, the corresponding SPADE plot is shown.

To investigate the metabolic changes along the hermaphrodite gonad arm, we constructed a new SPADE tree using only the pixels within the gonad region of the Up worm shown in the BCARS images and plotted the corresponding colored SPADE plots for each gonad section. See Figure 3 for the gonad schematic drawing and the corresponding colored SPADE plots and Figure S1 for an example of spatial segmentation of 10 sections in an image. The colored SPADE plots show the frequency of the pixels that are classified to the SPADE node, where dark blue indicates high frequency and yellow or green-yellow represents a relatively low frequency.

Figure 3 shows that neighboring sections share a similar pattern of nodes with a significant portion of high-frequency nodes in common, suggesting that their metabolic states are similar, and thus, their cell types are correlated. We observed that the high-frequency nodes concentrated at the center and middle branches for sections 1 to 6. In sections 8 to 10, the high-frequency nodes spread out from the center/middle branches to the left branch and the leaf nodes. In section 7 (the U-shape bend), the center, middle, and left branches are highlighted with high-frequency nodes, consistent with a known transitioning role within this section. The results of differential BCARS spectra (Figure 1e) support this finding, showing two major clusters of spectra. One cluster for sections 1 to 6 show strong positive peaks around 800, 1230, 1570, and 1665 cm^–1^ as well as negative peaks around 877, 1300, and 1450 cm^–1^. The spectra of another cluster for sections 8 to 10 appear the opposite sign for the values of the peaks mentioned above. These results are in agreement with the progression of germ cell development. Prior to the loop (section 7), the most significant cellular event is cell division, where germ cells undergo proliferation and differentiation for oogenesis. These germ cells maintain a connection to the cytoplasmic core of the gonad. After the loop (sections 8–10), the oocytes close the connection to the cytoplasmic core (cellularization) and expend themselves through yolk uptake for maturation.^47^ In the loop, both differentiated germ cells and growing oocytes coexist, where the meiotic germ cells programmed to enter apoptosis serve as nurse cells that contribute mRNA, protein, and cellular organelles through cytoplasmic streaming to the growing oocytes.^36^ These results demonstrate that the metabolic data collected by BCARS, after SPADE analysis, can be transferred into useful and biologically meaningful information for cell type discrimination.

**Figure 4:**
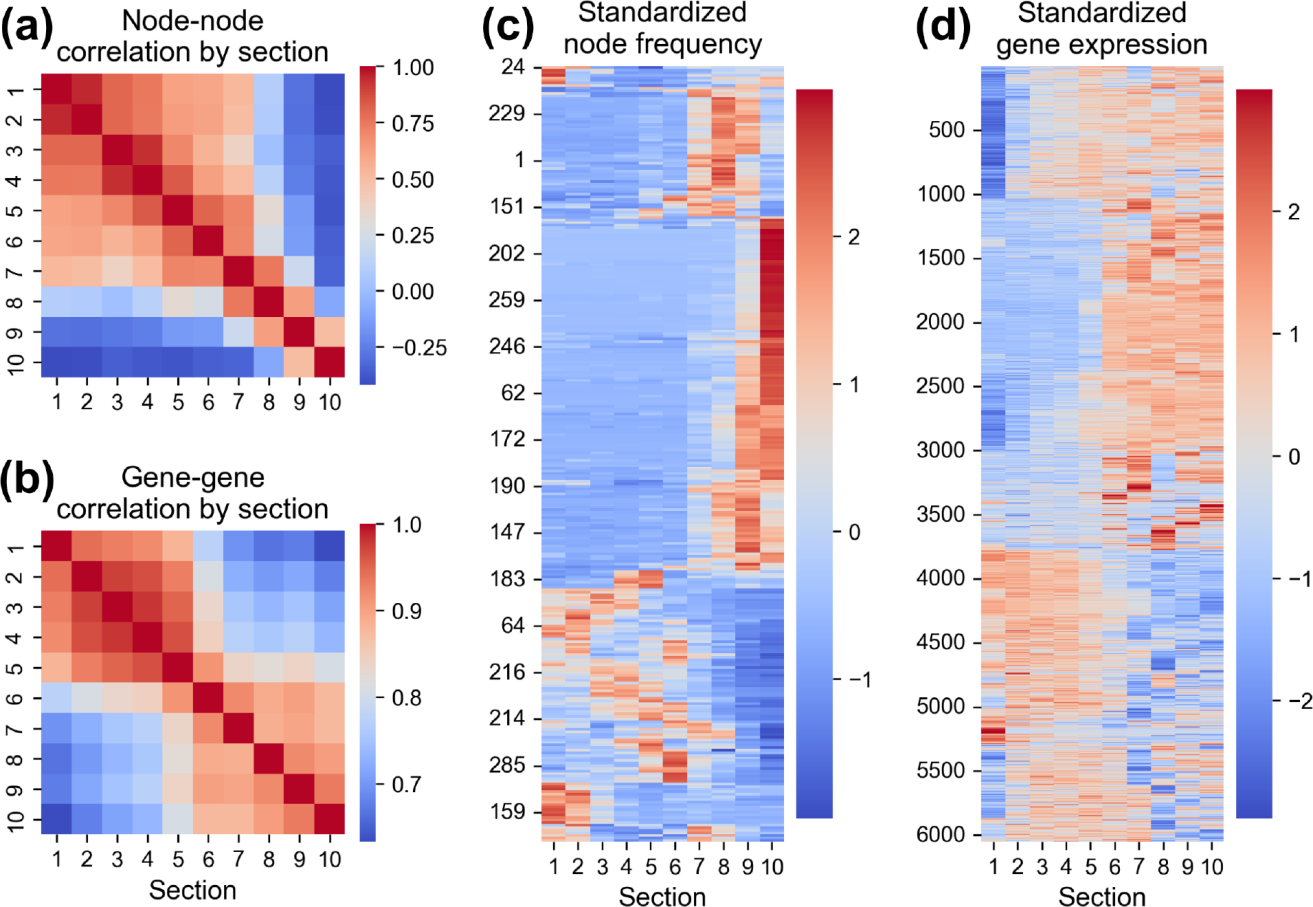
Comparison between BCARS SPADE results and existing gene expression data obtained by Tzur et al.^28^ (a) Correlation of Gonad tree node frequency between each pair of 10 sections of the Up worm gonad. (b) The same process applied to expression of dynamically expressed genes (genes that show 2-fold change in mapped read count) in the hermaphrodite worm gonad. (c) Node frequency data from Figure 3 reordered according to the clustermap generated in Figure S2. The color indicates the *z*-score standardized node frequency. (d) Same process applied to the dynamically expressed gene data, with the clustermap shown in Figure S3.

We next compared the BCARS results with the existing gene expression profile data^28^ along the hermaphrodite gonad (see the Materials and Methods section for the details of correlation and clustering analysis). As shown in Figure 4a,b, we found a similar heat map pattern between BCARS node–node correlation and gene–gene correlation plots. These plots were generated by correlating the vector (from each section) representing either the node probabilities or dynamic gene expression levels, respectively, against the corresponding vector from each of the 10 sections. While the gene–gene correlation plot shows a high correlation for the regions of sections 1–5 and sections 6–10, our BCARS results also show two major highly correlated blocks but with about 1 section shifting (sections 1–6 and sections 7–10). The difference is likely due to the temporal delay between gene and metabolite expression. Considering the causality of gene–protein expression and that *C. elegans* germ cell development is a continuous and fast process, with a high ovulation rate at 1 egg every ∼24 min per gonad arm, the time difference between gene–protein expression is projected in a spatial shift between gene and metabolite correlation patterns, which is likely ∼1 section shift shown in Figure 4a,b. Notably, because of the high sensitivity and high spatial resolution of BCARS, the contrast or the dynamic range shown in BCARS node–node correlation plot is stronger than the results obtained by the spatial transcriptomic approach. The dynamic range using the SPADE analysis of the spectral distribution is even greater than section-averaged mean spectra or difference spectra (Figure S7a,b).

As shown in Figure 4c,d, the heat maps of the standardized BCARS node frequency and standardized gene expression show a similar trend. We performed clustering analysis for the BCARS SPADE nodes (see the Materials and Methods section for details) and gene expression data that were previously reported^28^ and observed the same shifting (1 section difference) of the transition section when comparing the two heat maps. We also found that many SPADE nodes strongly correlated to the gene expression profile along gonad sections, suggesting that these nodes could indicate the spatial distribution of the gene-regulated metabolites in the BCARS images.

To verify this, we examined several genes critical for germ cell and embryonic development. These genes include *rme-1* that encodes a conserved Eps-15 homology (EH) domain protein RME-1, which is required for normal endocytic recycling and meiotic maturation of germ cells,^48,49^*plp-1* that encodes Pur-alpha-like protein-1 (PLP-1), which is essential for germline gene-silencing machinery,^50^ and a conserved collagen XVIII homologue gene found in *C. elegans* called *cle-1* that encodes the CLE-1 protein, which is necessary for tissue organization and structural integrity. The loss of CLE-1 function causes embryonic lethality and egg-laying defects.^51^

**Figure 5:**
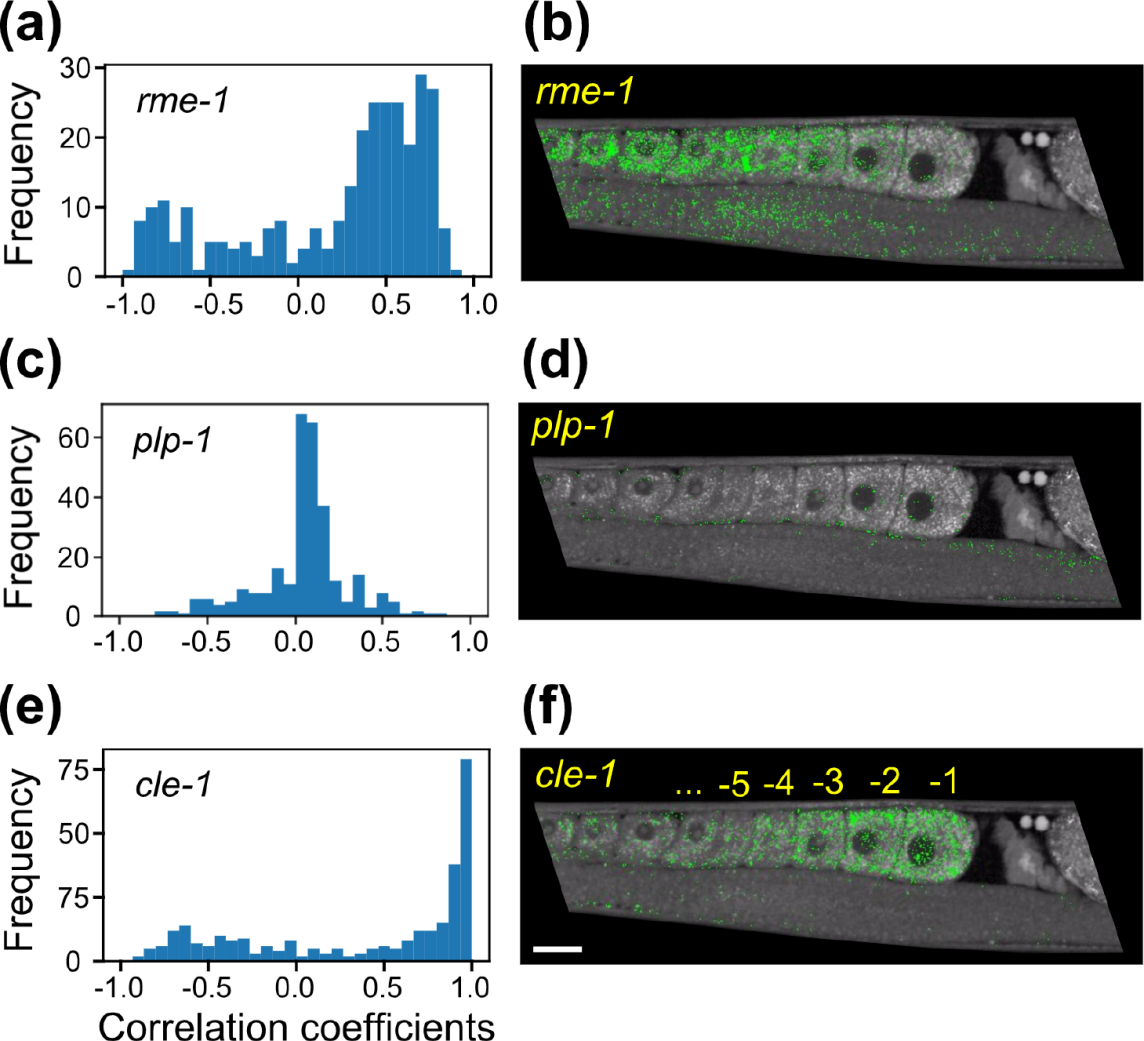
Spatial distribution of gene-correlated nodes. Panels a, c, and e are the histograms of *rme-1*-, *plp-1*-, and *cle-1*-correlated nodes, respectively. The 10 nodes with the highest correlation to the respective gene were chosen for the next step. Panels b, d, and f are the spatial distribution of corresponding gene-correlated node pixels (green dots) in the 2925 cm^–1^ BCARS image. Scale bar: 20 μm.

Figure 5 shows image data consistent with the idea that SPADE nodes with high correlation to these genes of interest could be used to spatially localize the downstream metabolic effects caused by activation of those genes. The expression level of the gene of interest in each section was correlated to the section fraction of each node in the Gonad tree. The histogram of the correlations of the nodes is shown in Figure 5a,c,e. The 10 nodes with the highest correlation to the gene were selected, and the pixels assigned to those nodes were highlighted in the BCARS images (green dots in Figure 5b,d,f). We found that the *rme-1*-correlated pixels are concentrated in the gonad sections 7–9, showing a positively correlated, consistent pattern similar to the spatial distribution of the *rme-1* gene expression (Pearson’s correlation coefficient >0.6, Figures S8a and 5a,b). In contrast, the number of *plp-1*-correlated pixels, as a negative control, is significantly lower, and the distribution of those pixels is relatively even throughout the whole gonad (Figure 5d). This is because the PLP-1 protein initiates the germline gene-silencing pathways, functioning as a protective mechanism that silences the expression of foreign genetic elements and suppresses the expression of many endogenous genes, with *plp-1* mutants showing 1367 genes with significantly higher expression levels without the protection of the germline gene-silencing mechanism.^50^ While the value of *plp-1* expression level is about 3 times higher than that of *rme-1* and remains approximately constant within the whole gonad,^28^ none or very few metabolites related to *plp-1* are produced and can be detected by BCARS, which is in agreement with the negligible correlation between BCARS SPADE nodes and *plp-1* expression profile (Figure 5c). Finally, we examined the *cle-1* gene. We found that many SPADE nodes are highly correlated to the regulation of this gene from the histogram of BCARS SPADE nodes (Figure 5e), and consistently, a very high positive correlation (Pearson’s correlation coefficient >0.9) for the spatial distribution between node pixels and the gene expression level is displayed (Figure S8b). An obvious, the progressively increasing number of *cle-1*-correlated pixels from the −5 to −1 oocytes suggests accumulation of *cle-1*-related chemical components in the maturing oocytes (Figure 5f). We thus characterized the BCARS spectra of those green pixels shown in Figure 5f. While the mean spectrum of *cle-1*-correlated pixels is similar to that of *rme-1* (Figure S8c,d), we found after subtraction of a reference spectrum, i.e., the mean spectrum of the whole gonad, the Raman features stood out (Figure S8e,f). Compared to the *rme-1* (Figure S8e), the differential spectrum of *cle-1*-correlated pixels (Figure S8f) shows two strong peaks at ∼850 and ∼940 cm^–1^ which are the marker bands for collagen.^33,52^ Other Raman features such as peaks at ∼1080 cm^–1^ (lipids), ∼1280 cm^–1^ (amide III), 1400–1500 cm^–1^ (lipids), and ∼1665 cm^–1^ (amide I) are all associated with extracellular matrix (ECM),^52^ consistent with the major biological role of the CLE-1 protein, which is one of the key proteins producing ECM molecules that support the structural integrity of tissues as well as the development of embryos.^51^ In addition to ECM, other subcellular localization results include lipoprotein granules that are identified primarily in the postloop region (Figure 2d) and phospholipid membranes (Figure S5), which are both likely to be correlated to specific gene families in the transcriptome. It requires further functional tests to verify those gene candidates and examine the change of the subcellular localization of the gene-correlated pixels. Nevertheless, these results demonstrate that BCARS can capture metabolic data from live, intact samples. The extracted metabolic information can be further correlated to specific gene expression profiles and, more importantly, be used to predict the possible distribution of gene-regulated metabolites at subcellular resolution.

## Discussion

We provide compelling evidence that spectroscopic coherent Raman imaging can map overall metabolic profiles with sufficient specificity to indicate gene regulation activity. In this demonstrative work, we found the minimum-spanning tree aspects of SPADE useful for visualizing the chemical progression accompanying phenotype changes. We also found that the population of spectral space reflected the gross similarities and differences in gene activation profiles among gonad locations and suggested a temporal (spatial) delay in metabolic response to some transcriptomic activity. Finally, we provide evidence that BCARS can map metabolite change profiles responsive to specific transcriptomic activity at subcellular resolution in live animals.

We anticipate that the applications of high-content metabolomics by BCARS microscopy could extend far beyond the germ cell maturation demonstrated in this work. It could additionally be used for rapid label-free classification in live cells, animals, or clinical tissues at subcellular spatial resolution. As we have demonstrated, the spatially and temporally resolved BCARS spectra are amenable to spanning-tree analysis that can reveal hidden links between pathways in biological systems, which will be helpful for studies in developmental biology, stem cell differentiation, or intercell networking in specific microenvironments.

The capability of BCARS for label-free discrimination between metabolic states of living samples makes it an attractive method for mapping the basins of attractions during critical, irreversible transitions such as cancer formation or aging. Our work demonstrates that BCARS can detect signaling activity and further specify the area possibly affected by the signaling pathways and thus could be a powerful diagnostic tool. For the purpose of phenotype identification and enumeration, spectroscopic Raman microscopy may be a viable surrogate for multiomic measurements in some cases. Mapping Raman microscopy onto phenotypes would democratize the ability to measure and analyze biological systems holistically, possibly in real time and noninvasively. However, while our current work shows the possibility of high-content metabolomics with BCARS, further orthogonal characterization, such as functional tests and gene knockout or knock-down experiments, are needed to verify the reliability and specificity of this approach.

In addition to the need to further explore the biological robustness of the approach we have demonstrated, our results bring some operational issues to the fore. When imaging with an intrinsically *z*-plane sectioning approach such as BCARS, it is crucial that either identical *z*-planes be compared between samples or that the full volume of the sample being imaged. This issue is exemplified in Figure S9a, showing the Gonad trees from Figure 3, which were trained on the Up worm, but now colored using data from Down, the other worm from the same image. The Down trees are strongly similar to the Up trees in sections 7–10, but only weakly similar in sections 1–5. (Section 6 of Down is not visible in the captured image and is hence excluded from the analysis.) Additionally, the coloring for section 3 is also different from all other sections. This is visible in the node–node correlation by section across worms shown in Figure S10a. The differences in sections 1–5 between Up and Down arise largely because these two worms were imaged in different *z*-planes, as is visible in Figure S4a–c. The part of the gonad before the loop contains nuclei arranged as a cylindrical shell. In Up, the *z*-plane was closer to the center, but in Down, it was off from the center. This causes nuclei to occupy most of the imaged area before the loop in Down, but only a small proportion in Up. Therefore, while the constituents of this volume are essentially the same (as shown in Figure 2a–d), their proportions are different, leading to the qualitative but not quantitative similarity in SPADE trees of sections 1–5 across the two worms. Section 3 appears to include two nuclei that should not be present at that location in the gonad; these may be nuclei from the gonad’s nearby sheath cells that may have also been captured. These issues can be resolved by full volumetric imaging of the organism. For the images presently under discussion, the worm gonad is 20 μm thick. Each *z*-slice corresponds to a thickness of 1 μm, so a full volumetric scan would take ∼25 slices, or ∼12.5 h. However, if a smaller area is examined, such as just the final egg cell (40 × 40 μm^2^ in size), the volumetric scan should take significantly less time (∼30 min in this case).

We have not investigated the long-term phototoxicity of imaging to the animal. We note that Chen et al.^53^ examined zebrafish embryo brain development under broadly comparable conditions (0.91 nJ per 140 fs pulse at 110 MHz, against our 0.12 nJ per 16 fs pulse at 40 MHz) for 20 h continuously and observed normal development into larvae in all of the several tens of individuals studied.

Another issue is subtle but apparently significant variation in retrieved Raman spectra between images. We use the intrinsic NRB to normalize Raman spectra retrieved from the BCARS signal. In practice, for control samples, we see intrasample variations on the order of a few percent. Here we show that resolving this minor variability may be crucial to implement a robust spatial metabolomics approach. To illustrate this issue, the SPADE classifier from Up was used on another worm from a different image which we label “One” (Figure S4d–f). Figure S9b shows the same trees as Figure S9a, but shaded using data from One. These trees look only vaguely similar to the Up or Down trees, with most of the data being projected onto a small number of nodes. This projection error suggests systematic differences between the two images. Small variations in retrieved Raman spectra due to slightly different imaging conditions could result in projection errors when a classifier is trained on one spectral data set and used to classify another. In Figure S11 and the associated discussion in the Supporting Information, we show that these artifacts can be largely resolved using difference spectra in the analysis.

## Conclusions

We have shown that BCARS can acquire spatially resolved Raman spectra from live *C. elegans*. We show that the variations in these spectra report on overall metabolic profiles and correlate strongly with known transcriptomic profile changes. We provide evidence that our observations are robust, suggesting that this approach may be successfully developed for use with other organisms. We believe such a tool based on BCARS could be extremely useful, enabling organelle-resolved and longitudinal gene-linked metabolomic studies that are now impractical or impossible.

## Data Availability

The hyperspectral images, analysis code, and documentation are available on GitHub at https://github.com/rajaspoorna/jpcb-spade-cri

## Supporting Information Available

Supplemental figures provide details of worm segmentation, overall gene–gene and node–node Seaborn clustermaps, worm images at multiple wavenumbers, additional cluster spatial distributions similar to Figure 2, dynamic range comparisons between the SPADE method described here and other Raman spectral analysis methods, spatial gene expression, SPADE analysis from Figure 3 for Down and One worms, spatial (sectionwise) correlations between SPADE trees for the three worms, and UMAP (instead of SPADE) analysis of the spectral data for all three worms showing distinct spectral changes across every worm section as well as the effectiveness of using difference spectra in reducing projection artifacts; a table with the number of spectra that were used in each section of each worm (PDF)
