# Correction to “Toward Gene-Correlated Spatially Resolved Metabolomics with Fingerprint Coherent Raman Imaging”

**DOI:** 10.1021/acs.jpcb.3c05167

**Published:** 2023-08-09

**Authors:** Rajas Poorna, Wei-Wen Chen, Arno Germond, Peng Qiu, Marcus T. Cicerone

**Affiliations:** † Department of Chemical and Biomolecular Engineering, Georgia Institute of Technology, Atlanta, Georgia 30332, United States; ‡ Department of Chemistry, Georgia Institute of Technology, Atlanta, Georgia 30332, United States; § Department of Biomedical Engineering, Georgia Institute of Technology, Atlanta, Georgia 30332, United States; ∥ UR 370 Animal Products Quality Unit, INRAE, Centre Clermont-Auvergne-Rhône-Alpes, 63122 St Genès Champanelle, France

Dr. Arno Germond is added as a coauthor of the article referenced above. Dr. Germond provided significant assistance in writing the grant that supported this paper and provided significant input into the early stages of project planning. We regret the oversight.

We also specify that Wei-Wen Chen and Rajas Poorna share first authorship. Both contributed equally to the paper. This information was specified in the original submitted article but was lost in the editorial process.

These changes are reflected in this Correction.

