# Supplementary material for: Toward Gene-Correlated Spatially Resolved Metabolomics with Fingerprint Coherent Raman Imaging
Source: J Phys Chem B. 2023 Jun 13;127(25):5576–87. doi: 10.1021/acs.jpcb.3c01446 (PMC10316396; doi:10.1021/acs.jpcb.3c01446)
Supplement: Supplementary file 1 — jp3c01446_si_002.pdf [file jp3c01446_si_002.pdf]

Towards Gene-Correlated Spatially Resolved Metabolomics with Fingerprint Coherent Raman Imaging

Rajas Poorna,[†,‡] Wei-Wen Chen,[¶,‡] Peng Qiu,[§] and Marcus T. Cicerone[*,¶]

†*Department of Chemical and Biomolecular Engineering, Georgia Institute of Technology, Atlanta, GA, 30332, USA*

‡*These authors contributed equally to this work.*

¶*Department of Chemistry, Georgia Institute of Technology, Atlanta, GA, 30332, USA*

§*Department of Biomedical Engineering, Georgia Institute of Technology, Atlanta, GA, 30332, USA*

E-mail: cicerone@gatech.edu

Phone: +1.404.894.2761

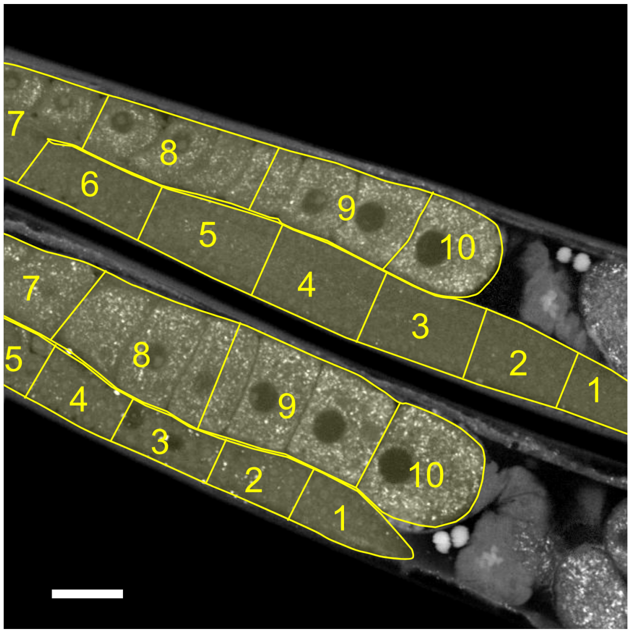

Figure S1: An example of spatial segmentation of 10 sections of the hermaphrodite gonad.

Table S1: The number of spectra comprising each section of each worm.

	Worm		
Section	Up	Down	One
1	3972	6055	2307
2	7381	5213	14035
3	8593	5183	15291
4	9678	5592	13210
5	9427	2591	11775
6	7682	N/A	8044
7	7876	7002	10162
8	13060	17376	7675
9	13114	19844	12778
10	8095	11805	12020

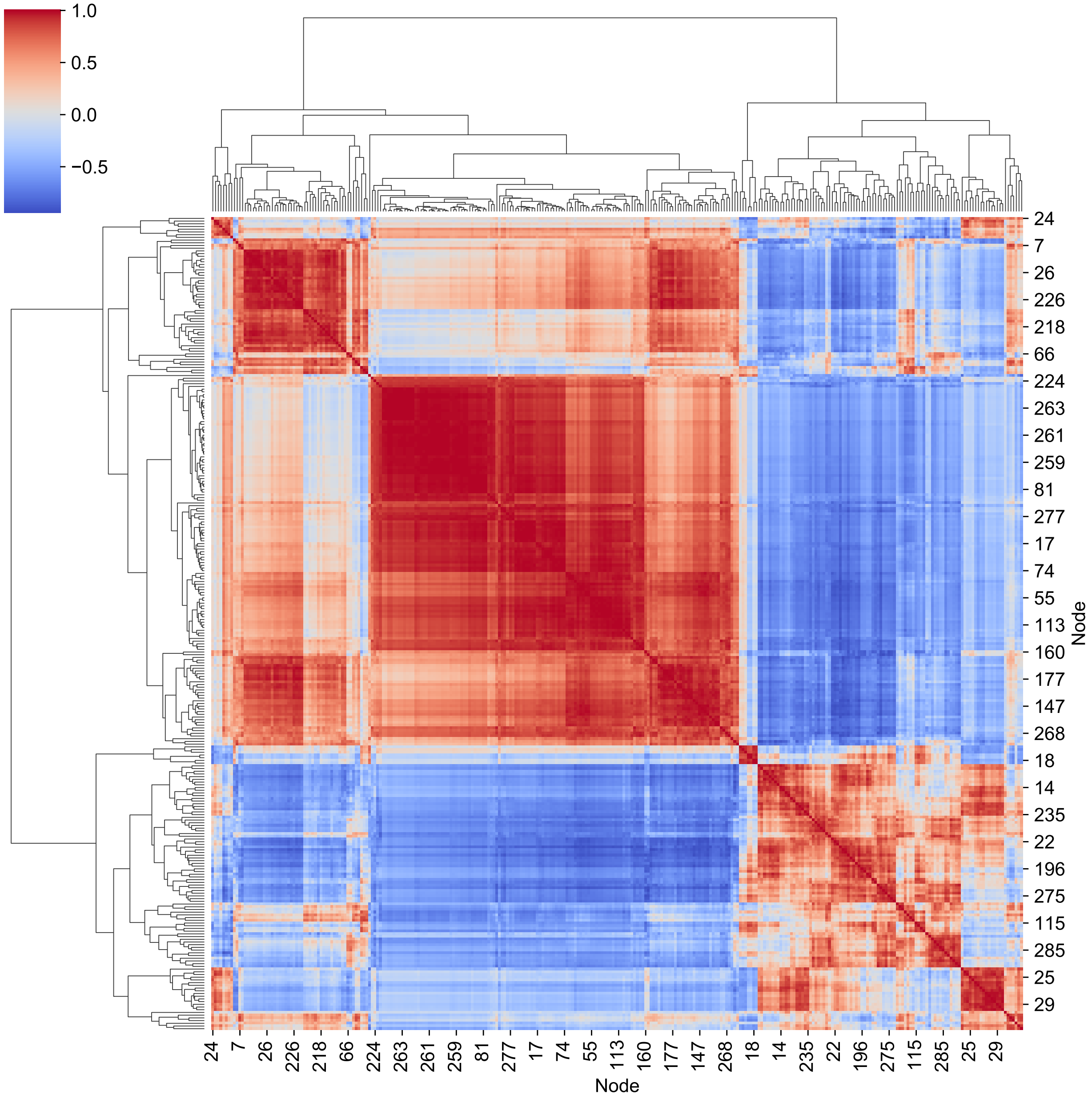

Figure S2: Clustermap generated from node frequency data from each section. Unlike in all other analyses, where the correlation was calculated between each section using node data per section (generating a 10x10 matrix), here, the correlation was calculated between each node. This 300x300 matrix was supplied to Seaborn's clustermap function to obtain this.

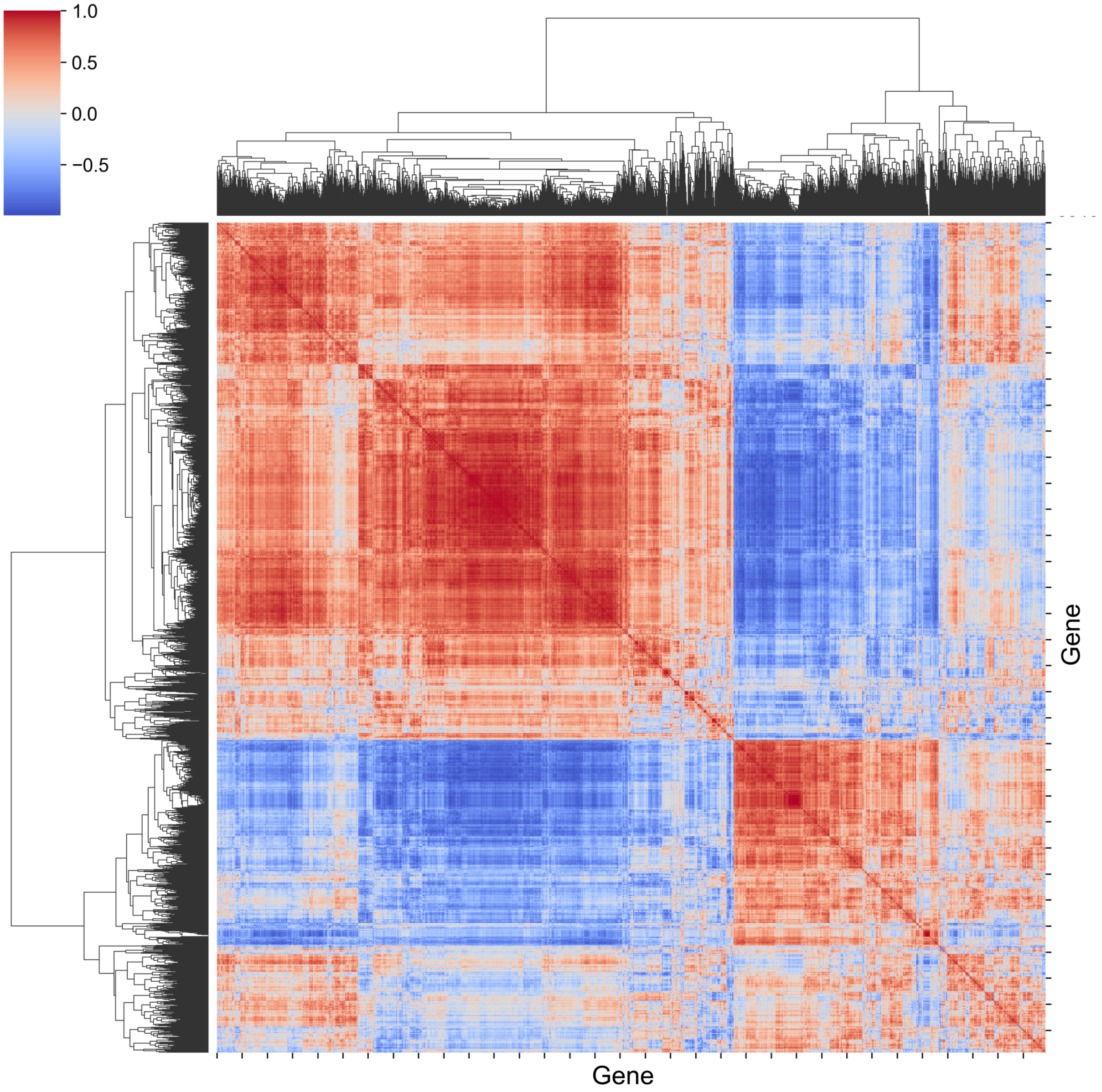

Figure S3: Clustermap generated from dynamically expressed gene expression data from Tzur et. al.[28] using Seaborn, with the same procedure as S2

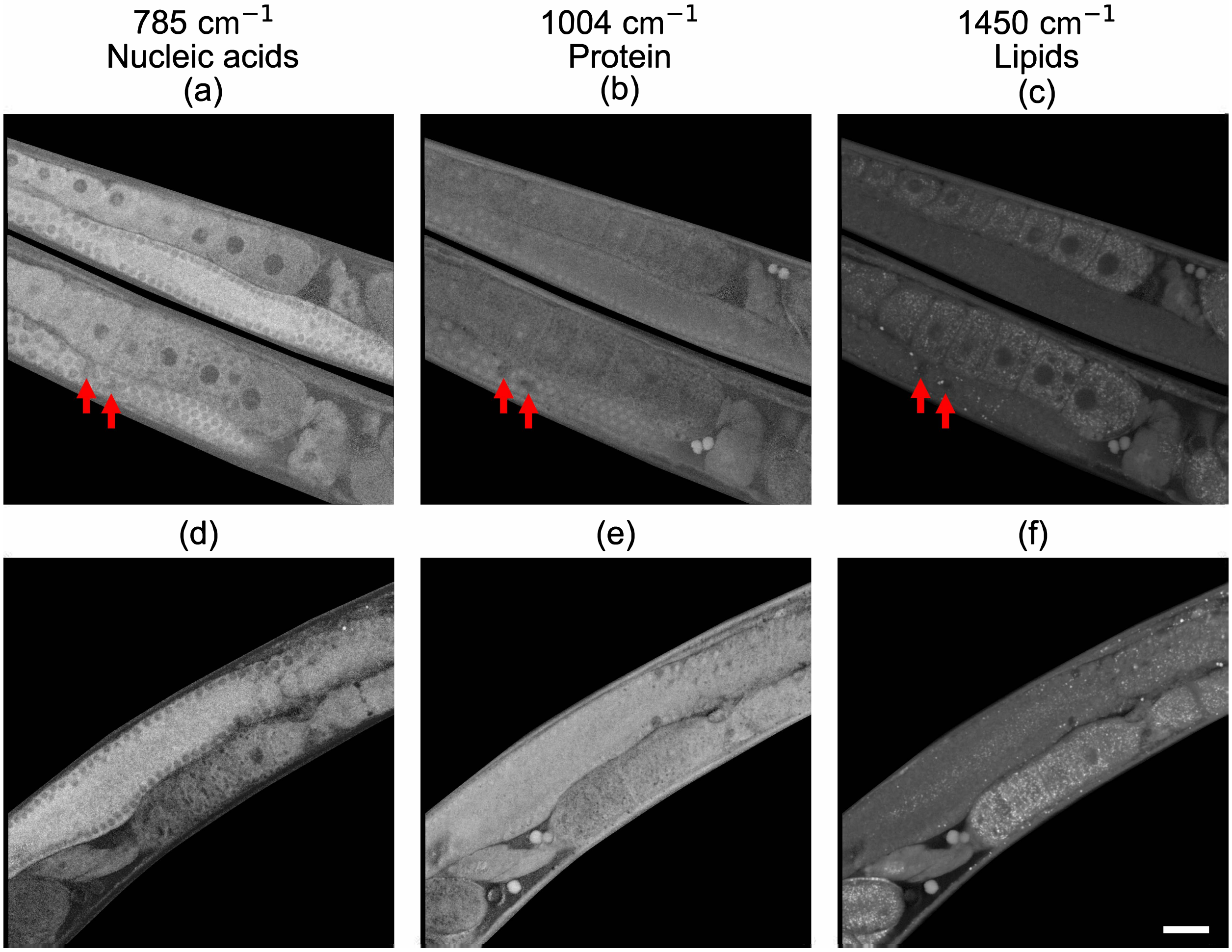

Figure S4: Worms imaged at multiple wavenumbers. The chosen wavenumbers specify the contrasts indicated above the images. Arrows point to mature nuclei that are not expected in the pre-loop gonad. These may be from sheath cells that were also imaged due to the finite z-plane depth of BCARS imaging. (a-c) Up and Down worms. (d-f) One worm. The scale bar (20 μm) shown in (f) applies to all images in this figure. Note that the contrast at 785 cm^{-1} is due to the vibration of the O-P-O phosphodiester bond. This wavenumber is the best-known marker band for DNA and RNA (nucleic acids), but phospholipids[37] and potentially other molecules that incorporate phosphate groups can also show some signal at this wavenumber. In (a), the large, more mature nuclei appear darker than the small nuclei. We note that both large and small nuclei are darker than their surrounding cytoplasm, likely due to the cytoplasm containing large amounts of RNA (and hence, O-P-O bonds). The large nuclei also incorporate more fluid, potentially as nucleoplasm, as suggested by Fig. 2(c), where the intercellular fluid branch also selects pixels within the large nuclei. Dilute fluid would contain less signal at 785 cm^{-1}, making these nuclei darker at this wavenumber.

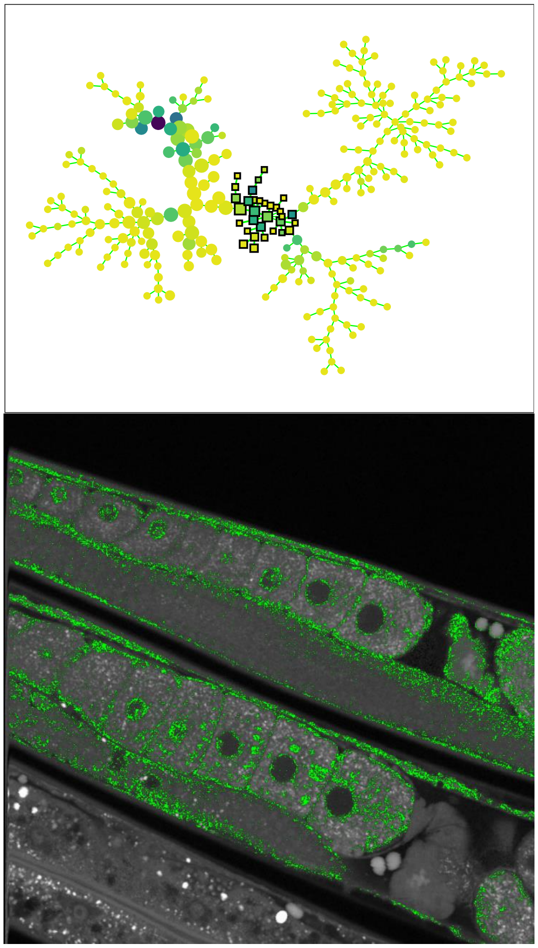

Figure S5: An additional cluster in the format of Fig. 2(a-d), depicting subcellular localization of phospholipid membranes.

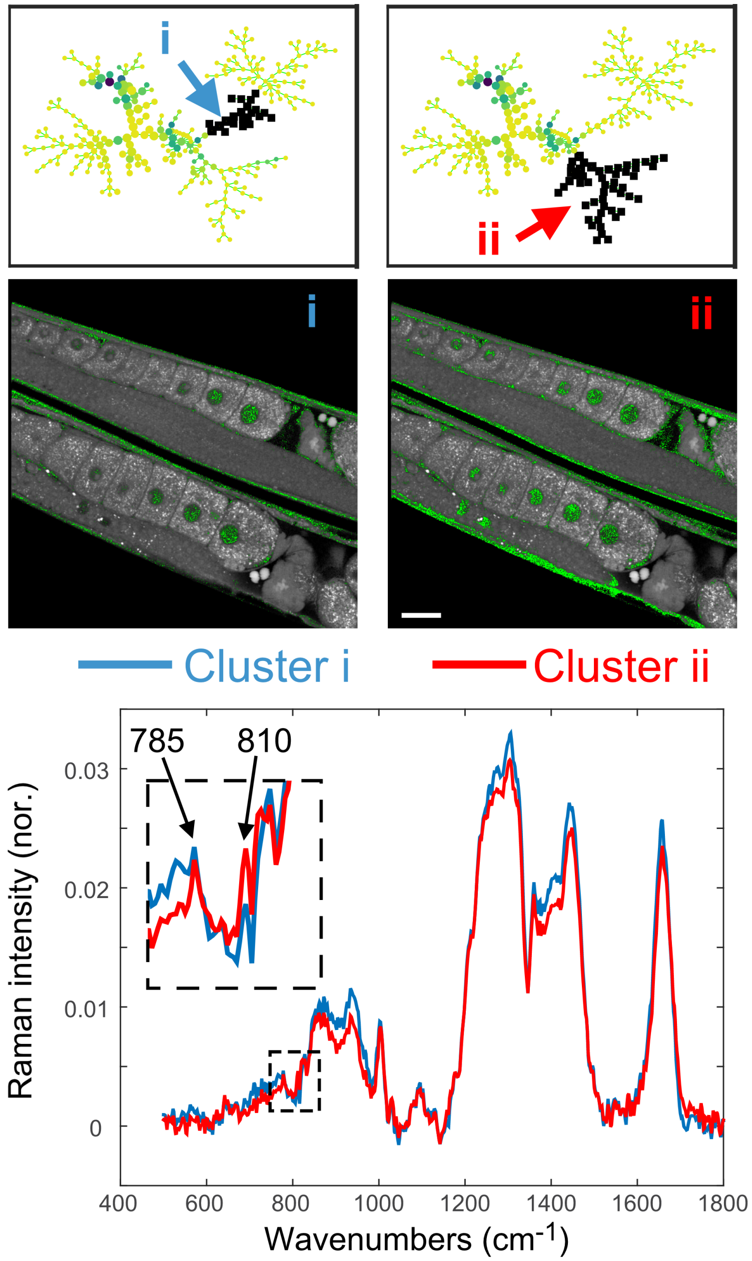

Figure S6: The clusters of nodes and their spatial distribution in the 2925 cm^{-1} BCARS image and the corresponding BCARS spectra. Cluster i and ii show similar spatial distribution but their Raman features are distinct. The inset panel shows the enlarged spectrum range around 800 cm^{-1}. Scale bar, 20μm.

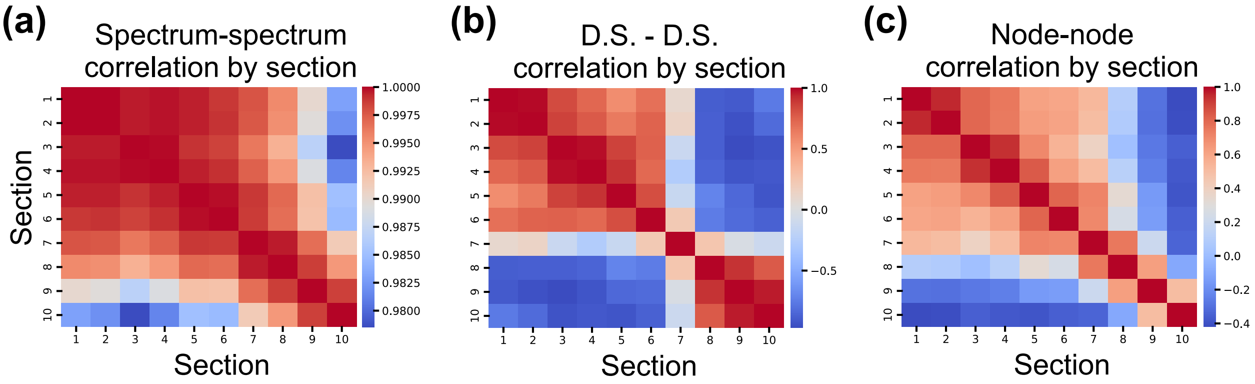

Figure S7: Comparing the dynamic range of various Raman spectrum analysis methods. The cells are undergoing a presumably continuous maturation as they pass through the gonad. Therefore, as the cell state resolving power of the analysis improves, we expect the section-section correlation matrix to approach a diagonal matrix with 1 on the diagonal and -1 in other locations. (a) Directly correlating the spatially averaged mean Raman spectra across sections gives the lowest dynamic range. (b) D.S. = "Difference Spectrum". Subtracting the mean spectrum of the entire gonad from each section average improves the dynamic range. Section 7 includes sections before and after the loop, and its mean spectrum is comparable to the mean of the whole worm. When the mean is subtracted, the difference spectrum is close to 0, leading to this exceptional behaviour. See Fig. 1e. (c) The SPADE analysis shows the highest dynamic range and cell state resolving power.

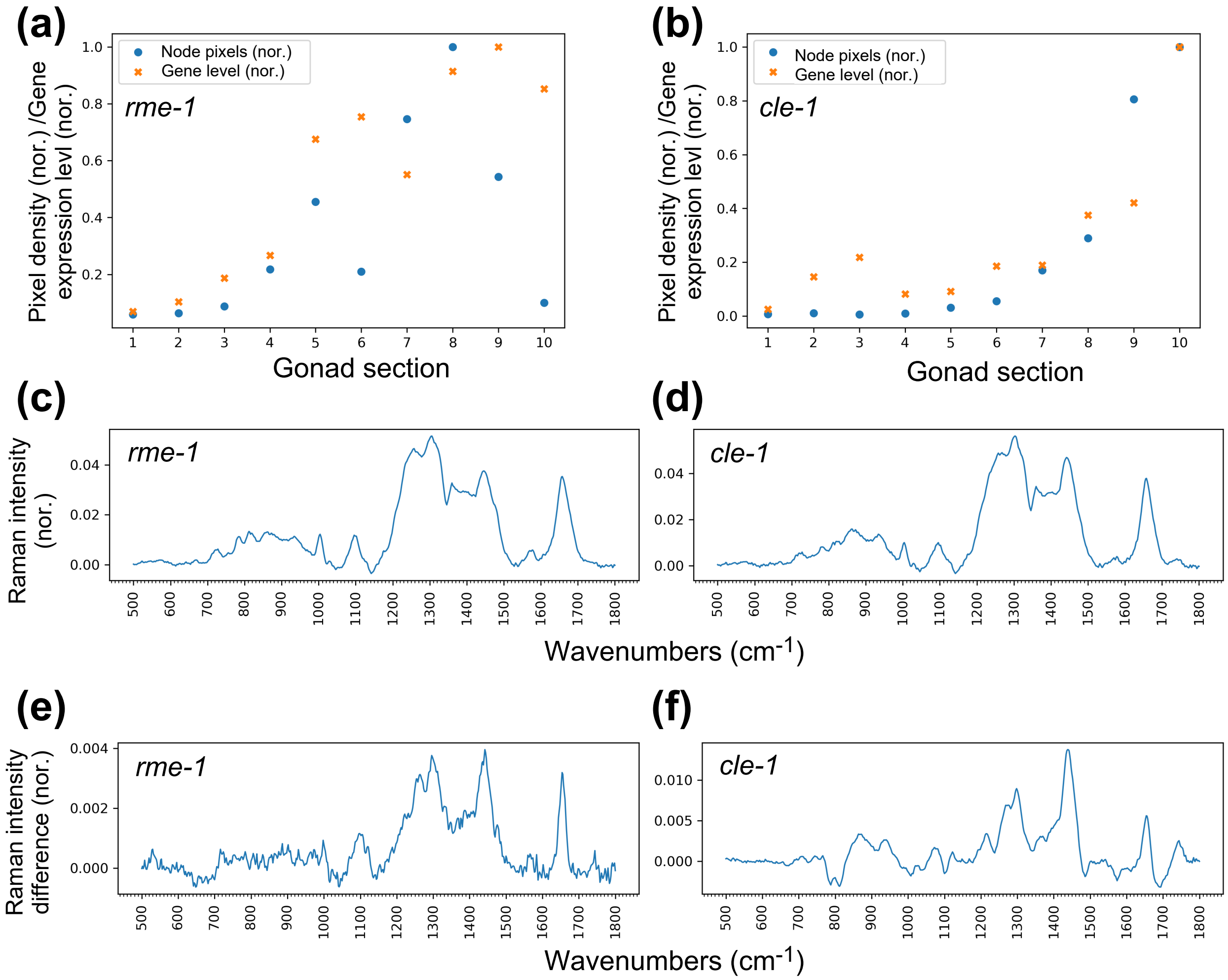

Figure S8: Gene-correlated node pixels and the corresponding BCARS spectra. The scatter plots of normalized gene-correlated pixel density and gene expression level by gonad sections for (a) *rme-1* and (b) *cle-1*. The corresponding mean BCARS spectra and differential BCARS spectra relative to the mean spectrum of the whole gonad for (c)(e) *rme-1* and (d)(f) *cle-1* correlated node pixels.

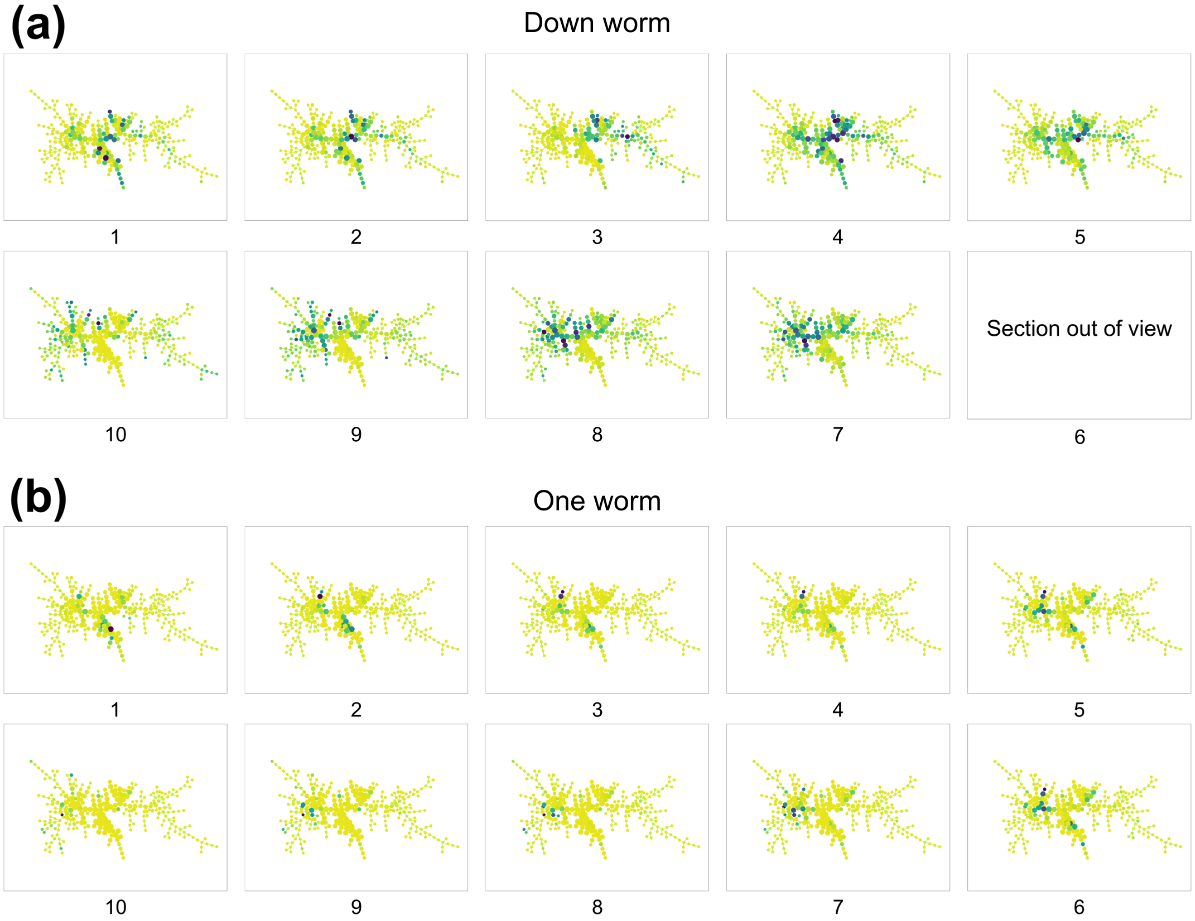

Figure S9: Coloring the Gonad SPADE trees from Fig. 3 using data from Down and One. (a) Down trees from 1-5 are shaded qualitatively similarly to those from Up, but the proportions are different, owing to a change in z-plane. Section 3 is different from all others due to the unexpected inclusion of two mature nuclei in the image (Fig. S4). Section 6 of Down is outside the image and hence excluded from the analysis. Sections 7-10 are very similar to those from Up. (b) One trees are only vaguely similar to the Up ones. While some similar nodes are highlighted, most pixels are assigned to a small number of nodes. This is characteristic of projection error, potentially due to systematic error in the retrieved spectra in the two images.

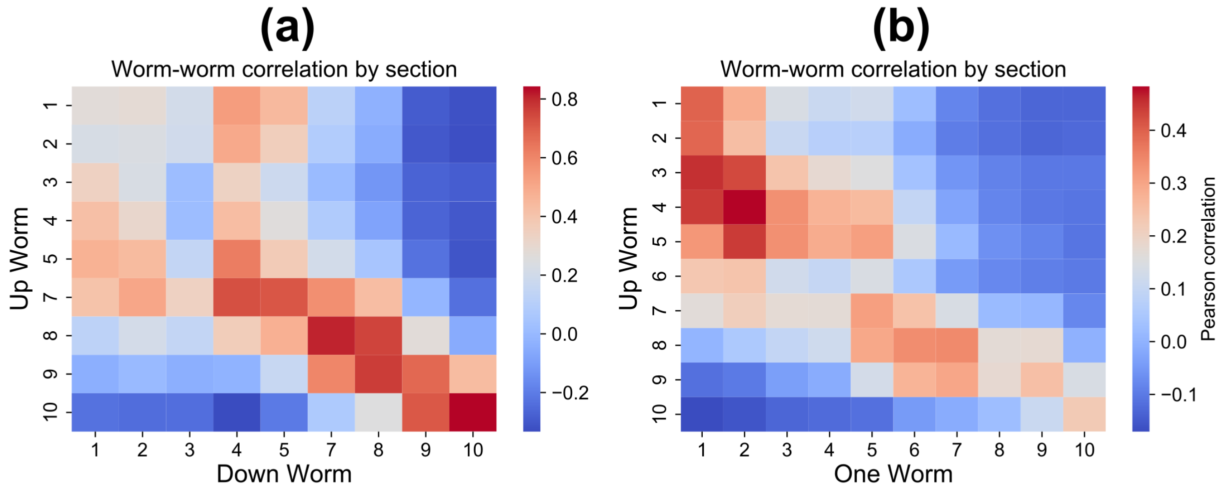

Figure S10: Comparing the Gonad trees highlighted by Down and One against Up (which was used for training the SPADE tree). This figure quantifies the arguments made in Fig. S9. (a) Correlating Up and Down worm Gonad tree node frequencies. (b) The same procedure applied to Up and One.

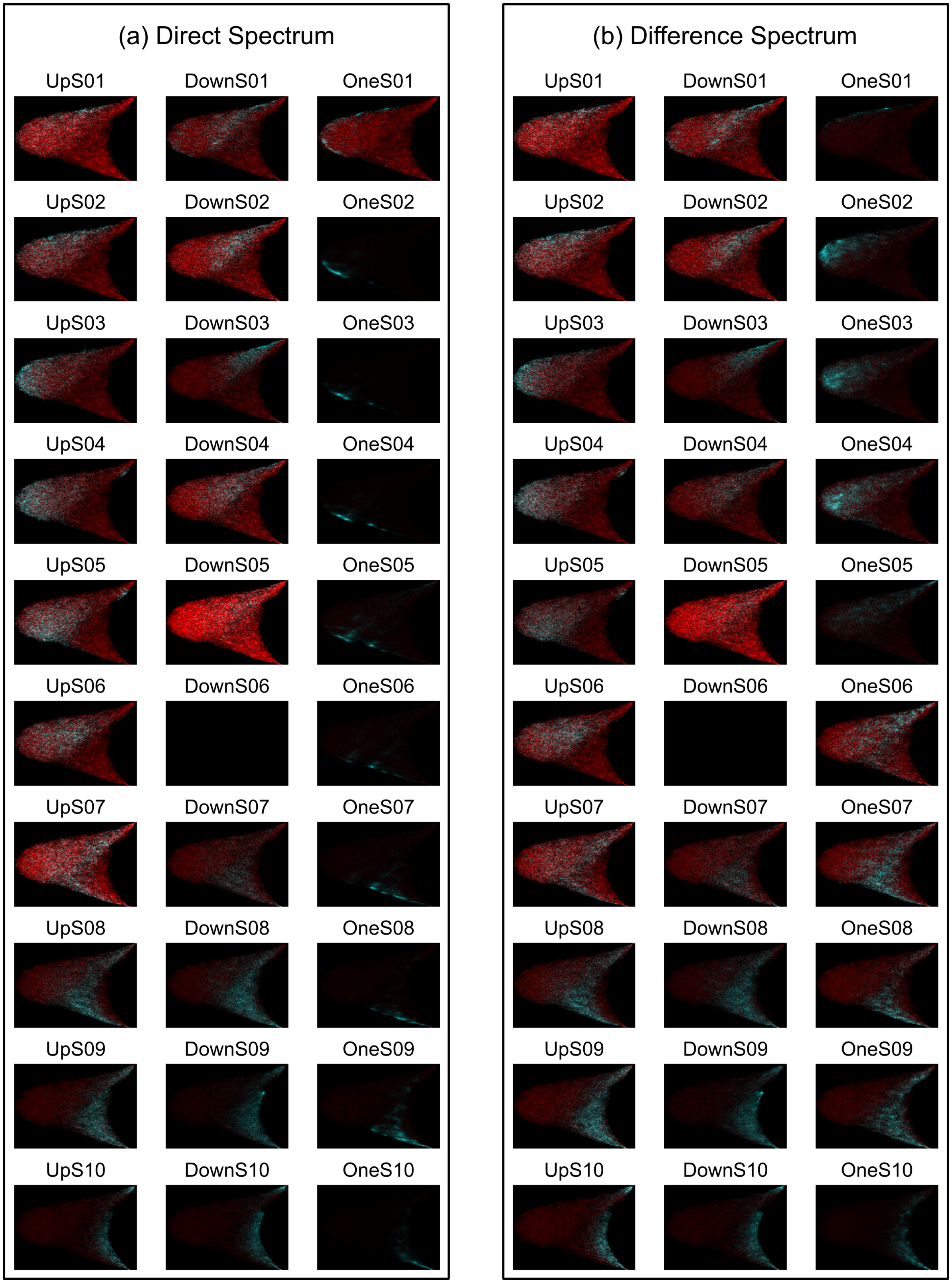

Figure S11: UMAP analysis of the three worms using the same procedure as SPADE. Comparison of projection error with direct BCARS spectra and difference spectra with the subtraction of an appropriate mean.

Figure S11 shows the same analysis performed for the gonads of the three worms using UMAP instead of SPADE. All spectra from the gonad of the Up worm were used to train two reducers: “Direct”, and “Difference”. For Direct, the spectra were supplied as-is. For Difference, the mean of the spectra in the gonad of the Up worm (or “UpGonadMean”) was subtracted from each spectrum supplied to UMAP. Thus, Direct reduces direct spectra, while Difference reduces difference spectra. The results of the two respective reducers are shown in (a) and (b). The Up, Down, and One columns show the results of reducing data from each section of the three respective worms.

The red, two-pronged shape in the background in both analyses is the respective training set (all the spectra from the gonad of the Up worm) represented using the respective reducer. The cyan pixels come from the respective gonad sections marked above each image. The intensity of the entire image is scaled according to the density represented by the cyan pixels. Thus, if the cyan density is extremely high, the red background becomes invisible, as in most images in (a) One.

Figure S11a, using the Direct reducer, shows the same kind of projection error as Fig. S9b in the One column, with most pixels being assigned to the same areas instead of being distributed across the graph. The overall trend while moving across the sections is qualitatively somewhat similar across all three worms, as before.

Figure S11b shows how difference spectra are much less susceptible to the projection error demonstrated by both SPADE and UMAP while reducing data from the One worm. Here, the UpGonadMean was subtracted from the spectra from both the Up and Down worms before reduction by the Difference reducer. However, for spectra from One, the “OneGonadMean”, the mean of the spectra in the gonad of the One worm, was subtracted from each spectrum before reduction. The One column now shows much less projection error, with pixels being spread out across the set, similar to Up and Down.

This suggests that the systematic error across images in BCARS is small and could potentially be corrected with simple modifications to the existing workflow, such as the use

of difference spectra.

Of note is that the UMAP reduction shows a continuous and significant change between every section in all three columns in (b), matching the intuition that the "cells" in these sections are undergoing continuous changes. A similar result was obtained when SPADE was performed with z-score standardized spectra (where the intensity at each wavenumber was mean-subtracted and is divided by its variance) instead of direct spectra (data not shown). This suggests that the BCARS microscopy data has much higher cell state resolving power than that shown in the analysis in this work.

The present analysis may be justified as follows. SPADE inherently ignores data density as information, due to the density-dependent downsampling step. UMAP inherently attempts to fit a manifold to equalise the data density on it. In other words, SPADE inherently discards data density information (density of points in spectral space), while UMAP exploits it. It is possible to "spread out" the data using techniques like z-score, so that SPADE is more sensitive to small changes in the Raman spectrum, while such a technique would have essentially no effect on UMAP.

However, when the z-score is applied, the branches in the SPADE tree are harder to interpret, and do not provide branches corresponding strictly to the well-understood cell components. This would complicate the analysis in Fig. 2. Future work can incorporate techniques to resolve this, such as annotating areas generated by UMAP using known information about the intensity and origin of Raman peaks at different wavenumbers, or a similar approach with SPADE nodes.
